# Further evidence that not all executive functions are equal

**DOI:** 10.2478/v10053-008-0004-5

**Published:** 2008-07-15

**Authors:** Christopher A. Was

**Affiliations:** Department of Educational Foundations and Special Services, Kent State University, Kent, Ohio, USA

**Keywords:** working memory, executive functions

## Abstract

The current study presents a comparison of 2 structural equation models describing the relationship between the executive functions of updating and inhibiting. Although it has been argued that working memory capacity is defined by one’s ability to control the focus of attention, the findings of the current study support a view of the executive control of attention that reflects updating and inhibiting as not entirely dependent on the same resources.

## INTRODUCTION

 In their original model of working memory (WM), Baddeley and Hitch (1974) proposed that the central executive controls the focus of attention and regulates cognitive processes. Later, Baddeley (1993) stated that he could quite easily have referred to his model as *working attention* due to the central executive’s control over the slave-systems, which maintain information through rehearsal processes, and the control of cognitive and attention processes. Baddeley and Logie (1999) acknowledged that WM is closely related to attention and that the central executive is often described as an attentional system. Baddeley (2000) commented that the Norman and Shallice (1986) supervisory attention system is a functional framework for describing the control of action and attention attributed to the central executive. Jonides, Lacey, and Nee (2005) hypothesized that storage and perceptual processing are mediated by the same brain structures, and that rehearsal in WM engages brain areas that also control attention to external stimuli. Similarly, Engle and colleagues have interpreted data gathered using traditional WM tasks to support their contention that working memory capacity is fundamentally related to the ability to control attention (see Engle, 2002, for a review): 

WM capacity is not directly about memory – it is about using attention to maintain or suppress information. WM capacity is about memory only indirectly. Greater WM capacity does mean that more items can be maintained as active, but this is a result of greater ability to control attention, not a larger memory store. Thus, greater WM capacity also means greater ability to use attention to avoid distraction (Engle, 2002, p. 20).

In other words, WM capacity is comprised of domain-general executive attention or control processes and domain-specific rehearsal and storage processes (Kane, Conway, Hambrick, & Engle, 2007). Essentially, WM span tasks measure controlled attention plus, short-term memory. These perspectives point to the importance of executive attention in WM. For example, Kane et al. (2007) contend that the executive attention processes that contribute to WM capacity are a significant contributor to fluid intelligence. 

Cognitive functions frequently attributed to the central executive, often referred to as executive functions (EF), include planning, decision making, abstract thinking, cognitive flexibility, and the inhibition of inappropriate actions. Recent additions to executive functions proposed by Baddeley and colleagues include temporary activation of long-term memory and shifting between tasks (Baddeley, 1996), and selective attention and inhibition (Baddeley, Emslie, Kolodny, & Duncan, 1998). Although all of these processes are attributed to the central executive, the current investigation contends that the specific executive functions of updating and inhibiting are not defined by a general ability to control attention.

Miyake, Friedman, Emerson, Witzki, Howerter, and Wager (2000) reported an individual difference study that supported the separation of executive functions into three categories: shifting, updating, and inhibition. Shifting refers to the back and forth switching between multiple tasks, mental sets, or operations (Monsell, 1996, as cited in Miyake et al., 2000). Updating is described by Miyake et al. as more than simple monitoring and coding of working memory representations but that “the essence of updating lies in requirement to actively manipulate relevant information in working memory, rather than passively store information” (Miyake et al., p.57).

Finally, inhibiting involves the deliberate suppression of automatic or dominant response patterns. For example, in the original color naming task (Stroop, 1935) when the color name and text color are incongruent, the task requires that the dominant response of saying the word be suppressed so that the goal response of naming the color of the text can be exhibited. From the descriptions of updating and inhibiting above, it seems necessary to determine if these processes are controlled by the same attention controlling processes.

Recent evidence suggests that not all EFs are related to higher cognitive processing in the same way. In a study of 234 twins, Friedman, Miyake, Corley, Young, DeFries, and Hewitt (2006) found that inhibiting, shifting, and updating tasks related to intelligence tasks in significantly different ways, suggesting that current measures of intelligence do not capture the range of EF. In a study of 11 and 12 year old children, St. Clair-Thompson and Gathercole (2006) demonstrated a bifurcation of executive functions using exploratory factor analysis. Although these researchers utilized measures of inhibiting, shifting, and updating, shifting did not emerge as a factor. The authors discuss this discrepancy between their study and the Miyake et al. (2000) of three separate executive functions. It is the contention of St. Clair-Thompson and Gathercole (2006), that the executive control necessary for successful completion of shifting tasks is not completed developed in 11-12 year olds, and therefore did not emerge as a factor in the studied sample.

The current study contends that the tasks that require the storage and updating of information (updating tasks) in the cognitive workspace are not completely dependent on one’s ability to attend to relevant information and inhibit irrelevant information, but that the two capacities are correlated yet separate. In a series of three experiments, Persson, Welsh, Jonides, and Rueter-Lorenz (2007) determined that the central executive is composed of separable mechanisms and that higher cognitive functions are dependent on limited resources. In the currents study, the comparison of two structural equation models (SEM) tested the hypothesis that inhibiting and updating represent distinct capacities. More precisely stated, the analysis of the data permitted a test of whether or not covariances in individual differences in tasks designed to measure updating and inhibiting executive functions can be explained by assuming one or two latent factors.

## Method

### Participants

One hundred eighty eight participants (132 females, 48 males, 8 not reported; mean age 25.7, range 18-56) received course credit in an introductory educational psychology course for their participation. These 188 participants were part of a larger study in which 270 participants received course credit for their participation. The tasks used in the current analysis are a subset of the tasks completed for the larger study. Due to attrition, several of the participants completed only one task of either the inhibiting tasks or only one of the updating tasks. Rather than estimate means and intercepts for participants who had completed only one task from each list, only participants who completed all six of the relevant tasks were included in the analysis of the current study.

### Materials and apparatus

Testing took place in a well-lit room containing six microcomputers. Participants performed the experimental tasks on IBM compatible microcomputers with 17” SVGA monitors and standard keyboards. Soundboard panels separated the microcomputers allowing for 1-6 participants to complete the tasks at a time. Due to the nature of the study, all participants completed the tasks in the same order. Data for the current study was collected as part of a larger study. Participants completed the tasks in five 1-hr sessions. Programming of all tasks was completed with E-Prime® software (Schneider, Eschman, & Zuccolotto, 2002). E-Prime® controlled the stimulus presentation, timing, and data collection.

### Design and procedure

Three measures of updating and three measures of inhibiting were used for the current study. The first WM measure was the alphabet WM task. In this task, participants performed 18 trials. Each trial began with the presentation of either one or two nonadjacent letters from the alphabet for 2.5 s, followed by a transformation direction and number (-3, -2, -1, +1, +2, +3). Participants were instructed to increment or decrement each stimulus letter according to the transformation value. The transformation value remained on the screen until the participant was ready to respond. When ready, the participant pressed the spacebar and saw eight response options. They were given 10 s to choose an option by pressing a number key form 1 to 8. Participants were instructed to complete all transformations before pressing the spacebar because of the short response window. This was done to prevent participants from solving the problems while examining the alternatives in the response window. Accuracy feedback was provided following each trial.

The 18 trials occurred in two blocks of nine trials. The trials of each block represented a 2 x 2 x 3 design with number of stimulus letters (1 or 2), forward or backward recoding direction, and recoding distance (1, 2, or 3) as the design facets. The order of trials within each block was randomized for each participant.

In the second updating measure (ABCD WM) each of the 18 trials consisted of the participants interpreting three aurally presented statements that together defined the order of the letters *A*, *B*, *C*, and *D*. One statement defined the order of *A* and *B* (e.g., “B comes after A”; interpreted as “AB”). Another statement defined the order of *C* and *D* (e.g., “D comes before C”; interpreted as “DC”). The third statement defined the order of *A* and *B* relative to *C* and *D* (e.g., “Set 1 comes after Set 2”; interpreted as “Set 2 Set 1” or “DC AB”). The ordering of the three statements and the ordering operations in each statement was varied across trials. Processing time for each statement is self-paced with a limit of 20 s. After all three statements are interpreted, participants select a response from an alphabetized list of eight possible orders. The 24 experimental trials were divided into two 12 trial blocks.

The third updating task, constructed for a use in the Was and Woltz (2006) study (numeral strings audio WM), is similar to the digit span backwards task but adds linguistically complex processing demands during retention of digits. In each trial, participants were presented aurally with six digits at a rate of 2.25 s per digit. Then participants answered two separate questions presented visually one at a time about the order of the numbers (e.g., if the digit string was “9 2 4 8 3 5”, the questions might be: “What number precedes 3?”, “What is the difference between the first and last numbers?”). All answers were numeric and participants entered them on the keyboard number pad. In the current study the dependent variable of interest in the analysis of the updating task was proportion of correct responses.

Three measures of inhibiting were used in this study. Two of the measures were adapted from Woltz, Gardener, and Gyll (2000). These two tasks represent a participant’s ability to overcome strong response tendencies that are in conflict with task goals. The first task, number disengagement, was developed using Posner’s principles of the attention-shifting paradigm (Posner, Snyder, & Davidson, 1980). The second task (number Stroop) is an adaptation of the original Stroop task.

In the number disengagement task each item presented in the practice trials was a large numeral from 1 to 9 (excluding 5) displayed in the center of the screen. The numeral was presented in black, 168 pixels (44.5 mm) wide by 227 pixels (61.1 mm) high on a 200 pixel (52.4 mm) wide by 400 pixel (104.7 mm) high white frame on a black screen. The participant’s task was to determine if the numeral was larger (greater than) or smaller (less than) five. Participants responded by pressing “L” for larger or “S” for smaller. Each of the 16 practice trials began with an orientation screen, which contained an asterisk in the center and lasting 1000 ms. A blank screen lasting 1000 ms followed the orientation screen and was followed by the stimulus. After responding participants saw a feedback screen regarding their accuracy. Feedback on accuracy and latency was also presented at the end of the practice block. The practice trials were designed to practice the participants at responding using the “S” and “L” keys.

Then participants were informed that the task would change and that the large numerals would now be formed from a pattern of smaller white numerals – text characters 10 pixels (2.6 mm) wide by 20 pixels (5.3 mm) high. Participants were told to continue to respond to the large numeral by pressing the “S” for smaller than five and “L” for larger than five. Participants performed two blocks of trials in this condition each block consisting of 12 facilitating stimuli (both large and small numerals greater than or less than 5) and four interfering trials (large numeral greater than 5 and small numeral less than 5 or large numeral less than 5 and small numeral greater than 5). Feedback on accuracy and latency was presented at the end of this practice block.

Next, the participants were told that the task was to change in an important way. They were informed that their task was now to respond to the small white numerals, not the large black numerals. Again, participants were told to work as quickly as possible while minimizing errors. Participants performed 48 random trials consisting of 36 facilitating trials and 12 interfering trials. Therefore, 75% of the trials required a response to the small numerals that matched the large numerals, to which participants are presumably practiced and attending. The interfering trials, accounting for 25% in this block, required the participants to disengage attention from the practiced mode of responding to the less practiced mode. Accuracy and latency feedback were presented at the end of the block.

The number Stroop task consisted of two parts. Part 1 consisted of two blocks of 20 trials in which participants pressed a number key corresponding to a single digit presented in the center of the display. The purpose of these trials was to practice the participants on using the four response keys with a single hand. Each block began with a warning to place four fingers of one hand on the number keys 1-4 at the top of the keyboard. Only the numbers 1-4 were used as stimuli, and they are presented in random order within blocks. Instructions emphasized response speed while minimizing errors. Following correct responses, latency feedback is provided for 1 s. After incorrect responses, the word incorrect is presented for 1s. Average latency was provided at the end of each block.

Part 2 was similar in format, except that character strings from one to four characters in length were presented, and participants were instructed to respond with the number of characters not the value of the characters. For each of four string lengths, there are five possible characters: 1, 2, 3, 4, and X. All characters within a string were the same (e.g., “33”, “XXXX”, “111”, “22”, etc.).

There were four blocks of 20 trials each in Part 2. Three different trial types correspond to those in the traditional Stroop task. Of the 20 trials in each block, 12 had content designed to interfere with the length judgment, (e.g., “2”, “3”, “4”, “11”, “33”, “44”, “111”, “222”, “444”, “1111”, “2222”, and “3333”). Four trials contained content designed to facilitate the length judgment (i.e., “1”, “22”, “333”, and “4444”). Finally, four trials contained content that is neutral with respect to length judgment (i.e., “X”, “XX”, “XXX”, and “XXXX”). Trial format and feedback are the same as described in Part 1.

A third task used in defining attention disengagement was a computerized version of the original Stroop color task (Stroop, 1935). Participants were informed in the instructions that this was a test of their ability to respond quickly to simple items and that each item would present a color name and their task was to press the corresponding color key on the keyboard. Stimuli consisted of the words “blue”, “red”, “green”, “yellow”, and a set of four Xs (“XXXX”) with each word being displayed in black, blue, red, green, or yellow. Participants then saw an example of the word “red” on the monitor display presented in black ink. The participants were then told to press the red key along the top row of the keyboard. They were then shown a second example of the word “blue” again presented in black. The participants were informed that they would complete a set of practice trials and asked to work as quickly and as accurately as possible.

Practice trials began with the instruction to “Get ready: Gently place your fingers on the colored keys on the keyboard.” This instruction remained on the display for 2500 ms. Next, a blank screen appeared for 1000 ms followed by an orientation screen containing an asterisk in the center of the display for 250 ms and then another blank screen for 250 ms. This blank screen was followed by the response screen containing the stimulus. After responding to the stimulus participants saw a feedback screen lasting 2000 ms that stated either “correct” or “incorrect” and an instructions as to the correct answer (i.e., “The correct answer was yellow, you should have pressed the yellow key.”) and ending the trial. After completing 24 practice trails a feedback screen displayed overall accuracy as percentage correct and the average response time per one trial. The purpose of these trials was to practice the participants on using the four colored response keys with a single hand.

After completing these practice trials participants were informed that the task would now change. The instructions informed participants that they would continue to see names of colors as before, but now their task was to respond according to the color in which the word was presented. Participants were then presented with two examples of stimuli, one in which the color name and the ink were congruent (e.g., the word “blue” displayed in blue), and one in which the color name and the ink were incongruent (e.g., the word “green” displayed in red).

Participants performed 10 practice trials consisting of 4 facilitating trials (trials in which the color name and ink were congruent), 2 interfering trials (trials in which the color name and ink were incongruent), and 2 neutral trials (trials in which the stimulus was four Xs presented in 1 of the 4 colors). After this block of trials participants again received accuracy and latency feedback.

Participants were then informed that the practice trials were complete and that the experimental trials were to begin. Again, participants were asked to work as quickly and as accurately as possible. Participants then completed two blocks of 60 trials each. Each block contained 24 facilitating, 12 interfering, and 24 neutral trials. Feedback on accuracy and latency was presented at the end of each block.

## Results

The first step in the analysis of inhibition data was the combination of latency and accuracy into a transformed adjusted response speed scores (see Woltz, 1990; Woltz & Was, 2007). Previous studies have found that the interference effect of the Stroop task is evident in both response latency (e.g, Stroop, 1935; Ward, Roberts, & Phillips, 2001) and accuracy (e.g., Kane & Engle, 2003; Rush, Panek, & Russell, 1987). As seen in Table 1, this pattern of interference was also demonstrated in the current study. Therefore, adjusted speed was computed for each task as the proportion of correct responses, divided by the average response time for all trials in the scale of minutes. Thus, the resulting speed scores are interpreted as number of correct trials per minute and are representative of a processing efficiency measure. One major advantage to this transformation, particularly for SEM, is that compared to response latency and error distributions, the adjusted speed distributions are closer to normal and the index has the advantage of incorporating meaningful variance of both latency and accuracy.

**Table 1.** Correlations Between Dependent Measures

Variable	1	2	3	4	5	6
1. Alphabet WM	.56					
2. ABCD WM	.31**	.82				
3. Numeral strings	.35**	.46**	.81			
4. Number disengagement difference	.21**	.12	.20**	.30		
5. Color Stroop difference	.22**	.13	.26**	.39**	.32	
6. Number Stroop difference	.38**	.19*	.25**	.41**	.45**	.36

Note. Values on the diagonal represent Spearman-Brown correlations between the firstand the second half of inhibition tasks,and the odd, and even number items on for updating tasks. * *p* < .05. ** *p* < .01.

The second step was to create difference scores from the speed scores of the inhibition measures. The differences of speed for the inhibition measures was calculated as a difference score between mean speed for interfering trials and mean speed for neutral trials (because the number disengagement task did not include neutral trials, the speed difference was calculated as the difference between mean speed for facilitating trials and mean speed for interfering trials). This measure represents a reliable measure of individual differences in the ability to disengage attention from the more attractive stimulus to the true response stimulus as accounting for simple reaction time. Although Spearman-Brown correlations between speed differences on the first and second halves of the inhibition tasks is not very high (see Table 1), the Spearman-Brown correlations between the neutral (or facilitating) trials in the first of half of the tasks and the neutral trials in the second half of the tasks was very high. This was also the case for the interfering trials (see Table 2).

**Table 2.** Spearman-Brown Correlations for Split-Half Reliability of Neutral and Interfering Inhibition Trials

Task	Neutral/Facilitating	Interfering	Difference
Number Stroop	.79	.67	.30
Number disengagement	.90	.54	.32
Color Stroop	.75	.83	.36

Table 1 displays the intercorrelations between the dependent measures for the six tasks (speed differences for inhibiting tasks and percent of correct responses for updating tasks). As state previously, in the current study proportion of correct responses was the dependent variable of interest in the analysis of the updating task, and the difference between the speed metric on neutral or facilitating trials and interfering trials, was the dependent measure for inhibiting tasks. Table 3 presents the mean and standard deviations for latency and accuracy of all six tasks.

**Table 3.** Mean Latency and Accuracy for Six Tasks

	Response latency (ms)		Accuracy (proportion correct)	
Variable	Mean	*SD*	Mean	*SD*
Alphabet WM	6564	2100	0,78	0,16
ABCD WM	2757	1088	0,87	0,16
Numeral strings	4732	1350	0,76	0,16
Number disengagement				
Interfering	791	204	0,95	0,06
Neutral	750	181	0,98	0,04
Mean difference	28	61	0,04	0,06
Color Stroop				
Interfering	943	187	0,94	0,09
Neutral	741	127	0,96	0,04
Mean difference	205	120	0,01	0,04
Number Stroop				
Interfering	693	115	0,94	0,05
Neutral	666	109	0,99	0,02
Mean difference	28	61	0,01	0,04

**Figure 1.**
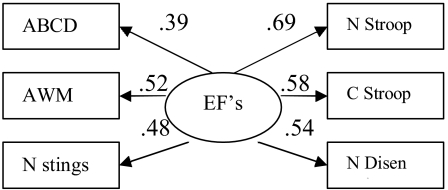
Model 1 with standardized parameter estimates. χ^2^ (9, *N* = 188) = 35.91, *p* < .001

**Figure 2.**
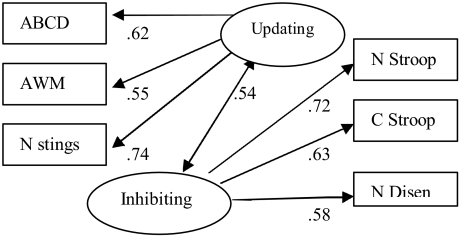
Model 2 with standardized parameter estimates. χ^2^ (8, *N* = 188) = 12.34, *p* = .137

Structural equation modeling (SEM) was used to compare two models. One that modeled WM as one factor constructed of all six tasks (Figure 1) and one model that described updating and inhibiting as two separate latent variables (Figure 2).

All parameters in both models were significant at ά = .05. However, Model 1 (see Figure 1) was not a good fit of the data, χ^2^ (9) = 42.50, *p* < .001; χ^2^/*df* = 4.72; *CFI* = .804; RMSEA = .141. Model 2 (see Figure 2) was determined to be a good fit of the data as indicated by the fit indices, χ^2^ (8) = 12.61, *p* = .13; χ^2^/*df* = 1.58; *CFI* = .973; *RMSEA* = .055. A chi-square difference test also indicated that that Model 2 was a significantly better fit of the data than model 1, χ_diff^2^_ (1) = 29.89, *p* < .001. Comparison of the two models supported the hypothesis that the process required for inhibiting are not the same as those involved in updating.

The structural equation models were also analyzed with data in which the updating tasks were also calculated using the speed transformation that was applied to the inhibition tasks. As in the first analysis, the one-factor model was not a good fit of the data, χ^2^ (9, *N* = 188) = 36.43, *p* < .001; *CFI* = .88; *RMSEA* = .136. The two-factor model was determined to be a good fit of the data as indicated by the fit indices, χ^2^ (8, *N* = 188) = 12.98, *p* = .113, *CFI* = .98, *RMSEA* = .058. A chi-square difference test also indicated that that when all variables were subjected to the speed transformation, the two-factor model was a significantly better fit of the data than the one-factor model, χ^2^ (1, *N* = 188) = 23.45, *p* < .001.

This second analysis was important to complete because participants were allowed to self-pace during the updating tasks. If it is the case that less able participants compensate for their poor ability by devoting more time to the task, transforming the updating data to the speed metric accounted for this latency-accuracy trade-off. Using the speed transformation for all observed variables in both latent factors did not result in any significant changes in the models, and the chi-square differences test between the two models was still significant. As stated, using the speed transformation for all tasks eliminated potential measure confounds created when latency is used to represent some constructs and accuracy is used to represent others.

## Discussion

Engle (2002) stated that his view of WM capacity as attention control, predicts that performance on the Stroop task depends on executive attention to maintain the goal of responding to the color in which the words are presented even when the written word elicits a stronger response tendency to respond to the name of the word. Maintaining the goal in an active state should be particularly difficult when some of the trials are congruent, that is the ink color and the word correspond. However, it should be harder to maintain the goal in active memory if the environment or context presents many trials on which performance can be successful without the necessity to maintain the goal to block the tendency to say the word. The findings of the current study agree with this contention. However, the current findings do not support the argument that the demands of updating are equally determined by an individual’s ability to maintain a goal in the focus of attention. The processes required for the completion of updating tasks have been compared to traditional WM processing. The processes involved in updating tasks (storage and processing) are virtually the same as those in traditional WM tasks and are often seen as measuring working memory capacity and not an executive function (Oberauer, Süß, Wilhelm, & Sander, 2007). St. Clair-Thompson and Gathercole (2006) found that measures of WM and updating loaded on one factor in a principal components analysis while measures of inhibition loaded on a second factor. 

In the current study, the comparison of the two models support the hypothesis that although inhibition is highly correlated to updating of WM, the resources available for specific executive functions might represent independent resources. At minimum, it is arguable that executive control of attention is not a unitary capacity. The analyses in the current study not only replicated those of previous studies (e.g., Miyake et al., 2000), but also expand on previous findings in an important way. Previous studies have focused on the relationships such as that between different executive functions and intelligence processes (Freidman et al., 2006), or have modeled the relationship between executive functions including shifting, inhibiting, and updating (Miyake et al., 2000). The current study explicitly focused on the relationship between updating and inhibiting because of the close relationship between updating tasks and measures of WM (St. Clair-Thompson & Gathercole, 2006) and because of the proposed close relationship between executive control of attention, as measured by the inhibition tasks, and the attention component captured in many WM tasks (Kane et al., 2007).

It is important to note that the variance not accounted for between the latent factors of updating and inhibiting might be based on one or more processes. It is possible that this variance reflects the processing components of the updating tasks. If this is the case, then some portion of updating processes are not accounted for by executive control of attention necessary for successful completion of the inhibiting tasks.

It is also possible the variance not shared between the two latent factors is based in the storage component necessary for successful completion of the updating tasks which could simply represent a short-term memory store. Engle (2002, p. 20) stated that “…WM is not about individual differences in how many items can be stored per se but about differences in the ability to control attention….” Although short-term storage is acknowledged as separate from the executive attention processing component of WM, it is an essential component in the completion of complex cognitive tasks, such as those in the updating tasks employed in the current study. Whether the unaccounted for variance represents, processing or storage it represents a process in complex cognitive processing that executive control of attention to a specific goal, as measured by the inhibiting tasks, does not explain. As demonstrated in the Freidman et al. (2006) study, these different executive tasks have distinct relationships with measures of intelligence. It is highly likely that is because different executive functions have different relationships with distinct complex and higher order cognitive processes.

An alternative interpretation is that the moderate correlation between the latent variables represented by inhibiting and updating tasks represents a higher order factor. This higher order factor might be interpreted as a general executive function resource. The current data does not allow for an analysis of a model containing a higher order factor because there are only two first order factors. This represents a limitation of the current study, and speaks to the necessity of further research of these constructs.

In either case, recent neural-imaging research also supports the separation of the different executive functions based on evidence that executive functions may be localized to separate portions of the prefrontal cortex (e. g., Smith & Jonides, 1997; Sylvester, Wager, Lacey, Hernandez, Nichols, & Smith, 2003; Wager & Smith, 2003). These neural-imaging studies, the research reviewed in this article, as well as the data presented in the current study, all support the necessity for research to model executive functions in relationship to complex cognitive tasks. This line of inquiry is important for the understanding of human behavior, education, and cognitive impairment.
